# Preserving independence among under-resourced older adults in the Southeastern United States: existing barriers and potential strategies for research

**DOI:** 10.1186/s12939-022-01721-5

**Published:** 2022-08-27

**Authors:** Ene M. Enogela, Taylor Buchanan, Christy S. Carter, Ronit Elk, Shena B. Gazaway, Burel R. Goodin, Elizabeth A. Jackson, Raymond Jones, Richard E. Kennedy, Emma Perez-Costas, Lisa Zubkoff, Emily L. Zumbro, Alayne D. Markland, Thomas W. Buford

**Affiliations:** 1 Department of Medicine - Division of Gerontology, Geriatrics, and Palliative Care, University of Alabama at Birmingham, 1313 13thSt. South, Birmingham, AL 35205 USA; 2 Department of Family, Community, and Health Systems, School of Nursing, University of Alabama at Birmingham, Birmingham, AL USA; 3 Department of Psychology, University of Alabama at Birmingham, Birmingham, AL USA; 4 Department of Medicine – Division of Cardiology, University of Alabama at Birmingham, Birmingham, AL USA; 5 Department of Medicine – Division of Preventive Medicine, University of Alabama at Birmingham, Birmingham, AL USA; 6 Birmingham/Atlanta Geriatric Research, Education, and Clinical Center, Birmingham VA Medical Center, Birmingham, AL USA

**Keywords:** Aging, Independence, Southeastern United states, Health disparities, Disability

## Abstract

Disability prevention and preservation of independence is crucial for successful aging of older adults. To date, relatively little is known regarding disparities in independent aging in a disadvantaged older adult population despite widely recognized health disparities reported in other populations and disciplines. In the U.S., the Southeastern region also known as “the Deep South”, is an economically and culturally unique region ravaged by pervasive health disparities – thus it is critical to evaluate barriers to independent aging in this region along with strategies to overcome these barriers. The objective of this narrative review is to highlight unique barriers to independent aging in the Deep South and to acknowledge gaps and potential strategies and opportunities to fill these gaps. We have synthesized findings of literature retrieved from searches of computerized databases and authoritative texts. Ultimately, this review aims to facilitate discussion and future research that will help to address the unique challenges to the preservation of independence among older adults in the Deep South region.

## Introduction

Preservation of independence is among the most important health priorities cited by older adults [1–3]. Undoubtedly, decades of research have supplied evidence regarding strategies to prevent age-related disability and maintain late-life independence. Still, many gaps remain regarding optimal strategies for maintaining independence among older adults at an increased risk for functional decline. For example, we recently highlighted the continued concern regarding disability among hospitalized older adults [4]. Although evidence exist on aging in place for persons dwelling in rural areas, relatively little evidence exists on strategies to prevent disability and maintain independence for older adults from rural areas and/or minority populations in the Deep South—despite extensive evidence surrounding disparate health outcomes among these groups.

In the United States, a key opportunity to improve the evidence base regarding independence and disability among older adults lies in increasing knowledge about those living in the Southeastern region also known as “the Deep South region”, a population commonly underrepresented in medical research. The Deep South states commonly referenced to include Louisiana, Mississippi, Alabama, Georgia, and South Carolina, typically rank near the bottom in the U.S. for a wide variety of health, economic, and access to care metrics. Moreover, approximately one-third of residents are from minority populations [5], and nearly one-quarter of minority residents live in poverty [6]. The most recent report from the Agency for Community Living [7] shows that most states with high rates of poverty for older adults (i.e., above 10%) are in the Deep South. Thus, the Deep South population represents a diverse population that suffers from broad health disparities, poor social determinant of health, and desperately needs more representation in aging and medical research in general. On the other hand, compared to other regions in the US, the Deep South is rich in culture, has a lower cost of living, strong family networks, has more green spaces, and has an increasing population growth [8–11].

Though the excess burden of chronic disease in the region is commonly recognized and elevated rates of age-related disability have been documented in the literature [12–14], relatively little work exists to document the precise etiologies and intervention strategies to address the unique contributors to age-related disability in the Deep South. The region [15] is unique in its culture and heritage, making it difficult to study certain cultural aspects of age-related disability in different regions. For example, adherence to the traditional “Southern diet” (e.g., culinary preference for fried food, high content of added fats, processed meat, and sugary beverages) was previously reported to increase the risk of stroke by 39% and mediated the Black-White racial disparity in stroke risk by 63% [16]. Thus, while stroke is a well-known contributor to disability among older adults, studies to address the impact of such localized risk factors on age-related disability are lacking. Moreover, the Deep South region has also suffered from a legacy of discrimination and medical experimentation that has resulted in a deep-seated distrust of the healthcare system as well as distrust of research for many in the African American community. These barriers have contributed to the vast racial and economic lines, highlighting the need for studies that incorporate the specific cultural and social conditions of the Deep South. Thus, the objective of this review is to highlight and assess knowledge gaps, as well as emphasize needs and potential opportunities to address age-related disability and independence research in older adults who are under-resourced and at increased risk for functional decline, focusing on the U.S. Deep South region as an example of where health inequality exists.

## Conceptualization of disability

**Fig. 1:**
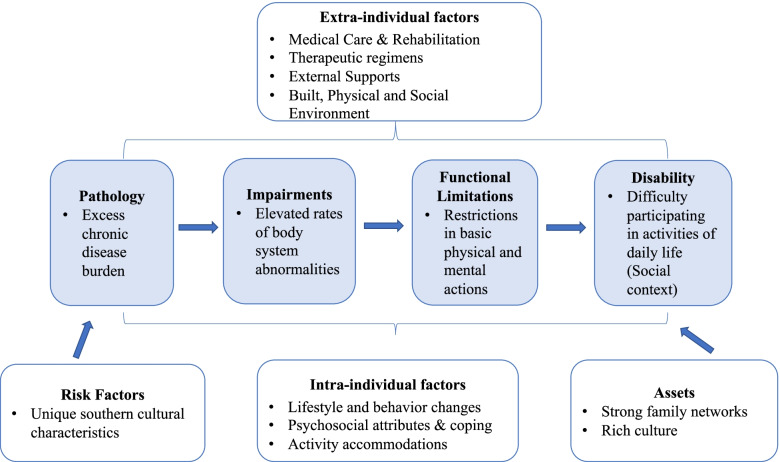
Adapted model of disability based upon work by Verbrugge and Jette [17] conceptualizing age-related disability in the Deep south

For the purposes of this review, we will frame disability largely on the work of Verbrugge and Jette [17], which combined the integral aspects of both the Nagi model of disability with International Classification of Functioning (ICF) model. We endorse this model as we concur that the two canonical models are complementary rather than competitive. In particular, the view of disability and participation as socio-cultural concepts [18], are especially relevant given the unique backdrop of the Deep South region. The inclusion of aspects of disability prevention and rehabilitation are also key to our conceptualization of independence (Fig. 1). We have the adapted the model of disability based upon work by Verbrugge and Jette [17] whereby we conceptualize age-related disability in a sociocultural context incorporating physical, mental, and social domains. The model includes three potential areas of disability—physical, mental, and social. These aspects of disability align closely with the World Health Organization’s definition of health as “a state of complete physical, mental, and social wellbeing”. Additionally, the National Institute on Aging’s life course theory focuses on “a multidisciplinary approach to understanding the mental, physical and social health of individuals, incorporating both life span and life stage concepts that determine health trajectory and influence population-level health disparities” [19].

**Fig. 2:**
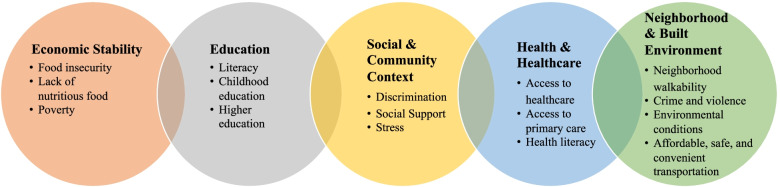
Categories and examples of social determinants of health [24]

For this review, we have defined these terms as follows: *Physical disability*—is related to physical function and includes declines in mobility, activities of daily living (ADL) or instrumental ADLs due to physical impairments. *Mental disability*—is broadly defined to include both mental health and cognitive impairment leading to declines in activity or participation. Mental disability can be seen as distinct from physical disability, as the people with dementia may have the physical ability to. For example, bathe themselves; still, their mental or cognitive impairment prevents them from completing the task. Finally, *Social disability*—for our purposes is defined as the degree to which older adults manage social roles. All humans have a number of different social roles they play, and they often switch between roles multiple times each day. Compared to activities of daily living or similar constructs, social roles are typically more difficult to assess as they are not necessarily performed day after day and some domains may be more subjective than others. Compared to physical and mental disabilities, significantly less is known about factors that impact social disability—such as the importance of social determinants of health (SDOH), health disparities and other environmental influences. Indeed, poverty and other SDOH can be both a cause and consequence of disability and may play an important role in social as well as physical and mental disability [18]. For example, poverty can limit access to healthcare and preventive services, increasing the risk of both catastrophic and progressive disease, making disability more likely. In addition, having disabilities increases the likelihood of experiencing material hardships, such as food insecurity, compared with people at a similar income level who do not have disabilities [18]. These social components are often not observable thus the term “invisible disability” could be used to describe these factors [20]. The consideration of SDOH (Fig. 2) is critical in the evaluation of health and disability, particularly in promoting health equity [21–23]. We utilize the focus on physical, mental, and social disability throughout this review to highlight key gaps and strategies to address age-related disability in the Deep South.

The Deep South region provides a unique opportunity and challenge to study the impact of SDOH on the prevention of age-related disability; unfortunately, to this day, studies directly evaluating the impact of these factors on age-related disability are lacking. Salient features that make the Deep South unique regarding SDOH include: 1) a low rate of adult literacy —estimated as 15%-16% [25, 26], and 2) high prevalence of poverty—i.e., Louisiana, Mississippi, and Alabama have poverty rates at or above 10% [27]; moreover, adding complexity, the specter of segregation and systemic racism continues to contribute to a variety of health disparities the region Therefore, the Deep South region not only provides a unique opportunity, but also presents a clear gap in knowledge regarding the interplay of SDOH, age-related disability, and maintenance of independence among older adults who are under-resourced and at an increased risk for functional decline. Below we further discuss potential barriers to research in the Deep South region that have contributed, at least partially, to the existing gaps in knowledge related to maintenance and loss of independence among older adults in this region.

## Barriers to be addressed

**Fig. 3:**
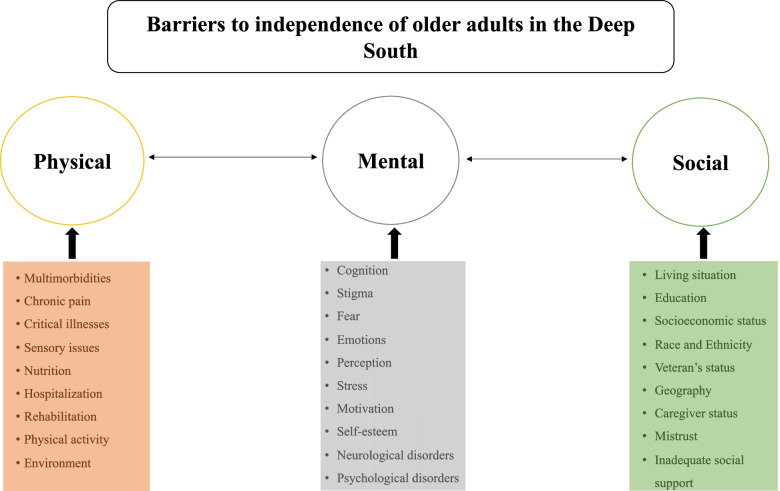
Physical, mental, and social barriers for preserving the independence of older adults in the Deep South

Undoubtedly, the barriers to be addressed in eliminating disparities regarding the quality of healthy aging are too numerous and expansive to be fully addressed in a single review; thus, here we focus on a concise overview of the challenges facing many older adults in the Deep South (Fig. 3). Several studies have indicated that African-Americans—a population overrepresented in the Deep South—are less likely to have access to quality care, as well as care in general, and more likely to suffer higher morbidity and lower quality of life [28–31].

Similar to disparities in access to quality care and poorer health outcomes, clinical trials have been under increased scrutiny for not having adequate diversity, including in sex and older age groups [32]. As a recent example, most racial and ethnic minorities and adults 65 years and older have been underrepresented in U.S. vaccine clinical trials over the last decade [33]. While this report excluded current COVID-19 vaccine trials in the analysis, a growing body of evidence exists that participants of most clinical trials are not truly representative of the general population, leaving gaps in understanding health disparities and increased burden of diseases. In the context of understanding health disparities for all clinical trials and not only vaccine trials, many studies did not include factors, such as social determinants of health (e.g., socioeconomic barriers), implicit bias, and an increased burden of comorbidities [34–37].

We posit that clinical scientists can improve healthcare equality by using the following guiding principles: 1) not conflating important identifying constructs (e.g., race/ethnicity; sex/gender), 2) recognizing, acknowledging, and understanding why significant variations within groups as well as between groups exist, and 3) incorporating our understanding of such differences to provide individualized care. We suggest a deeper and fuller understanding of culture that uses a socioecological framework (see Fig. 1) to explicitly integrate social determinants of health to facilitate the development of more effective and sustainable biomedical and behavioral interventions for maintaining function and treating disabilities in older age [38, 39]. In addition, these approaches likely would build trust between patients and families from varied backgrounds and the providers who care for them. Fewer clinical trials are available at hospital systems where people of color are more likely to receive care [40]. Existing inequities underlie the mistrust that many individuals from minority communities feel toward mainstream institutions and its practitioners. This mistrust stems from historical events, social inequalities, and social injustice related to medical care and research that occurred in the Southeast U.S [41]. The sources of this mistrust must be explicitly acknowledged, understood, and addressed before we can move forward in improving care for diverse populations with disability. This is similar to some recent recommended approaches for improving access to hospice and palliative care for minority communities where healthcare providers are encouraged to correct their own biases, build trust, and integrate varying value systems and approaches into care provision [42, 43]. 

### Potential groups of older adults in need of further research

Among population groups who are under-resourced and at risk for functional decline, older adults may have medical or social barriers that put them at risk for poorer functional outcomes. For instance, medically at risk groups refer to those groups of older adults with particularly acute risks to independence that stem from some medical/clinical issue. These types of challenges may be recognized in the literature, but in our view need significantly expanded research given their critical contributions to disability. Social barriers, often less recognizable, has insufficient research bases and is particularly acute in the Deep South. Notably, the conceptualization of physical, mental, and social disabilities may individually and jointly be present in either group of older adults at risk for functional decline.

### Hospitalized older adults

Older adults account for 40% of hospitalizations in the US despite being only 16% of the population. Additionally, 16.8% of older adults experience at least one hospitalization annually [42]. In 2018, the southern region had the highest rate for three or more days of hospital stay compared to other regions of the United states [43]. Hospitalized older adults are particularly at risk to a range of disabilities. Among hospitalized older adults with unplanned hospital admissions, the rate of mortality following discharge increased with age [44]. Physical disability in the form of loss of ADLs can occur within days of hospitalization, which has been termed “hospital-associated disability” (HAD) [45]. We recently reported that nearly one-third of older adults hospitalized for acute care developed HAD, with little change in rates over a 30-year period despite considerable change in hospitalization practices [4]. Prior studies have identified numerous pre-admission, hospital-associated, and post-discharge risk factors for HAD [46], with decreased mobility cited as at least one potential mechanism [47].

Cognitive disability is also a common occurrence among hospitalized older adults. A systematic review found that 12.9%—63.0% of this population has pre-existing dementia [48]. Approximately 10%-15% of hospitalized older adults exhibit delirium on admission and an additional 10%-40% are diagnosed with delirium during their stay [49]. Hospitalization is also associated with long-term acceleration in the rate of cognitive decline [50], at least some of this acceleration may be due to the occurrence of delirium during hospitalization, which has been associated with long-term cognitive decline even among patients who were cognitively normal on admission [51, 52].

Finally, concerning worse outcomes following hospitalization, health disparities exist among minority and rural-dwelling individuals. A recent study report that the risk of mortality or discharge to hospice was higher among Black patients compared to White patients [53]. Similarly, rurality was associated with greater in-hospital mortality rates even after adjusting for comorbidities and hospital-level factors [54]. Other disparities exist for hospitalization outcomes with SDOH underlying those differences. In one study, individuals with lower socio-economic status (SES) were more likely to die during hospital admission compared to those from higher SES classes [55]. Although the Deep South contains a large proportion of racial minority groups, rural dwellers and those with low SES, studies assessing the impact of these factors on aging are scarce. All three forms of disabilities interact and can affect hospitalization outcomes among older adults. Therefore, to improve aging-related outcomes, it is necessary to develop research that targets/incorporates the various forms of disabilities in this medically at risk population.

### Older adults with multiple chronic conditions

Multimorbidity, defined as the diagnosis of ≥ 2 chronic diseases, is estimated to affect approximately 64% of older adults aged 65 years and older, and accounts for a large proportion of overall multimorbidity diagnoses in the United States [56]. Common diseases associated with multimorbidity include, but are not limited to, the following: cancer, cardiovascular disease, hypertension, diabetes, chronic obstructive pulmonary disease, osteoarthritis, chronic pain, and kidney disease. The Deep South exhibits higher rates of multimorbidity compared to other regions in the United States [57]. Alabama and Mississippi report at least a third of the population in each state to suffer from multimorbidity, with older adults accounting for a large proportion of these reports [58]. Multimorbidity is associated with decreased life expectancy, or premature mortality [59, 60], thus highlighting the need for further understanding of socioeconomic interactions within the Deep South region. Therefore, it is necessary to target these barriers to alleviate the burden of multimorbidity that within this region.

Older adults in the Deep South are at an increased risk of developing multiple chronic diseases as a result of unique biological, economic, social, and environmental factors compared to other regions of the United States. The Deep South is comprised mainly of rural communities with a large proportion of the population being low-income households, having low levels of education, and with little access to health promoting facilities and education centers—all aspects correlated with an increased risk of multimorbidity [56, 61]. Low rates of physical activity, high body mass, and diets high in saturated fat and calorie dense foods, (as opposed to nutrient dense foods), are reported as prevalent within this region, and are all related to the increased risk of chronic low-grade inflammation with a subsequent increased risk of multimorbidity [62–64]. Age alone is a risk factor for multimorbidity with additional increased risk via gender and race. Women and non-Hispanic Whites are susceptible to higher rates of multimorbidity in the United States, but a shift towards non-Hispanic Blacks occurs in the Deep South—with Black adults reporting higher incidences of chronic disease compared to their non-Hispanic White counterparts in this region [65]. Understanding the interactions among each of these unique factors faced by older adults in the Deep South is imperative for decreasing the incidence of multimorbidity.

Decreased quality of life and increased rates of hospitalization are common in individuals with multimorbidity [66]. Multiple factors interact with multimorbidity and contribute to inauspicious outcomes. For example, polypharmacy, or the prescription of ≥ 5 medications, is common in individuals with multimorbidity and increases the risk of adverse clinical outcomes and harmful drug-to-drug interactions [67]. Additionally, polypharmacy in conjunction with multimorbidity is related to increased incidence of hospitalization and may be an indicator for mortality [68]. In addition to strategies targeting multimorbidity factors that are common across populations, the Deep South older adult population will greatly benefit from strategies that are specifically catered to their specific needs and such strategies may help reduce burden of multimorbidity in this population.

### Older adults suffering from chronic pain

Chronic pain appears to disproportionately affect older adults, such that the prevalence of persistent pain climbs steadily with advancing age until at least the seventh decade of life [69]. It is estimated that by 2050, 36.3% of the U.S. population will be over 65 years old, and the number of people ≥ 80 years old will more than triple [70]. Chronic pain is one of many pathological conditions that contributes to decreased independence of living [71]. Many forms of chronic pain are associated with a disability, which in turn promotes increased risk of mortality [72]. In older adults, chronic pain often leads to long-term social, mental, and physical deficits manifesting in other comorbid conditions such as obesity and depression [73, 74]. These patterns of deficits in independence could drastically exacerbate chronic pain.

Age-related chronic pain causes limitations in physical function, ranging on a scale of difficulty performing activities of daily living, such as walking, climbing stairs, and physical exercise [75, 76]. Additionally, chronic pain can result in absenteeism from social functions and unpredictable engagement in social outings that can lead to a decline in mental health [77]. Consequences can also extend to family and friends who become caretakers, which can lead to additional negative effects to the individual living with chronic pain.

Impacts of chronic pain extend across the US. However, chronic pain and resulting disability are potentially more common in rural areas, which are more prevalent in the Deep South compared to other regions of the US [78, 79]. Individuals from a lower socioeconomic background tend to experience greater pain severity and disability with chronic pain [80]. The biopsychosocial model of chronic pain highlights the interrelated associations among personal factors, such as age, SES, social, mental, and physical outcomes [81, 82]. However, consideration of these associations at a geographic level is lacking. We seek to highlight the need to examine these associations in older adults with chronic pain. Older adults with chronic pain face major challenges in overcoming pain-induced interference in social, mental, and physical abilities [83]. These challenges demonstrate the critical need for further investigation into age-related chronic pain. Specifically, future work is needed investigating the effects of chronic pain on the aging process and that expounds upon potential biopsychosocial differences contributing to pain disparities in the Deep South.

### Older adults from rural areas or with low SES

Successful aging is a multifaceted process that encompasses not only the physical and mental capacities of older adults but also the resources and additional support they have access to and utilize [84]. Though there are efforts to make healthy aging more prevalent, there are subsets of the growing older adult populations, namely in the Deep South, that are faced with financial struggles as a result of inequities of resources contributing to disparities in health outcomes [85, 86]. Successful aging is at least partially a function of SES—indicative of a person’s financial situation, educational attainment, and employment [85]. Generally, lower SES may lead to reduced access to healthcare, subsequent poor health outcomes, and an increase in morbidity and mortality with age [87–89]. These associations may be tied to a combined impact of increased stress, trauma, and limited access to appropriate healthcare [90–92], thus, contributing to excess burden of chronic disease and disability [85, 93]. An abundance of evidence exists highlighting, globally, the relation between SES and health outcomes, with the majority of studies showing that low SES is associated with barriers to healthcare access [88, 89, 94], and subsequent poor outcomes and death. In the Deep South, a unique combination of disparities may exist and exacerbate the relations between SES and health outcomes, including rurality.

Rurality also characterizes the Deep South and impacts the quality of life, particularly in older adults [95]. The term ‘rural’, according to the US Census Bureau, is any population, territory, or housing that is not a densely settled urban area [96]. Older adults living in rural areas are at a disadvantage due to lack of resources and services. This leads to an increased prevalence of chronic disease and disability and reductions in healthy behaviors [97–99]. Among older adults living in rural areas, the primary concerns that affect their health are typically access to healthcare and support, housing, and social isolation [97]. Approximately 10 million people (23%) aged 65 and older live in the rural US [100]. Of the states in the Deep South, Mississippi has the highest percentage of adults age 65 and older living in rural areas (55%), followed by Alabama (45%), South Carolina (36%), Georgia (32%), and Louisiana (30%) [101]. As previously mentioned, these states often have populations that are more diverse and older, though unhealthy and impoverished; thus, highlighting a need for understanding how their living in the Deep South affects their health.

A combination of poverty, age, racial/ethnic disparities, and poor infrastructure are challenges facing adults aging in the Deep South. Educational attainment is lower and those individuals in the rural Deep South face disadvantages regarding employment opportunities. Overall, these factors contribute, individually and synergistically, to the health of older adults living in the Deep South. In the past few decades, there have been national efforts focusing on addressing the health of the United States, most notably the Healthy People initiative. Two of the five overarching goals guiding the decisions of the upcoming “Healthy People 2030” U.S Department of Health and Human Services (DHHS) initiative are to “achieve health and well-being through the elimination of health disparities and achieving health equity” by “creating healthy social, physical, and economic environments” [102]. To achieve these goals, it is important that research targeting regions and populations that suffer from poor health outcomes is focused on bridging the disparity gap, in order to achieve health equity by addressing a critical need for more information on the health of this population through research.

### Older adults of racial minority populations

Early recognition of health disparities in racial minority populations indicates the need for both the outcome and the context within which health outcomes occur. The 1985 Report of the Secretary’s Task Force on Black and Minority Health explain in depth many of the current issues still affecting racial minority groups [103]. Access to health information, utilization and cost of healthcare, and availability of data and research, are still present-day challenges in providing optimum care for racial minority populations [103]. Older racial/ethnic minorities face additional barriers to maintaining independent living. As a group, older Black adults have been found to report significantly higher levels of disability than White adults [104–108]. Older African Americans consistently report diminished capacities to perform activities of daily living compared with other racial/ethnic groups [105, 107, 109]. In a study on a sample of African Americans from the Baltimore Study on Black Aging, Ayotte et al. found that body pain was significantly associated with ADL disability in both men and women and having two or more comorbid conditions was significantly associated with ADL disability in African American women [110]. ADL disabilities are also related to higher mortality rates, poor health outcomes and more cognitive decline in African Americans [110].

While previous research suggests that these higher rates of disability occur due to lower income and education attainment [106, 107, 109], there are additional social determinants of health, such as lack of access to quality health care [111, 112] and healthy food choices (“food deserts”) that underlie health disparities among racial minority groups. The onset of diseases also differs by racial groups in the United States. Since African Americans live longer periods of time with multiple undiagnosed/untreated chronic conditions, this might contribute to greater disability in this population [104, 113, 114]. Exploring factors such as these will shed additionally insights that can assist health professionals to develop interventions are developed to keep older adult minorities safe from injury and disability in their various communities. In fact, the exploration of medical conditions such as diabetes, depression, and pain and how these conditions disproportionality impact the daily lives of ethnic and racial minority individuals should be a major area of focus for future disability-related research [105, 108, 115].

### Older adults living as caregivers

Most evidence linking the terms “caregiving” and “older adults” together consists of a directional relationship that ties informal and formal care provision for older adults alone. Older adults can become caregivers for other family members, who are themselves older or incapacitated. Therefore, another relationship that remains to be explored includes older adults serving as caregivers and this can be itself, a form of social challenges. In the Deep South, between 2015 – 2017, the prevalence of caregiving among individuals aged ≥ 45 years was respectively, 26.2%, 24.5%, 23.9%, 22.7%, and 26.7% for Alabama, Georgia, South Carolina, Mississippi, and Louisiana [116]. In 2015, about 34% of all caregivers in the United States were aged 65 years or older [117]. These caregivers provide emotional support, assist with domestic chores and personal care, support with medical and health care needs, and guide decision making where the individual being cared for is not capable [118, 119].

In the context of the Deep South, there remains a substantial gap in research characterizing the nature, impact, and long-term effect of caregiving provided by older adults. Similarly, epidemiological data on the impact of caregiving roles on social challenges among older adult caregivers in the Deep South remains scarce. Current knowledge on family caregivers of older individuals is drawn from survey, but remains underpowered in assessing these determinants by racial subgroups or geographical regions [120]. Optimal healthcare services that take into consideration the various stressors and life needs of older adult caregivers should become a priority to policy development and intervention planning [121].

As older caregivers have other responsibilities, the caregiving role might present with social barriers that impact their health. The time commitment for caregivers of individuals with more than 2 self-care needs is comparable to two full-time jobs [122]. Older adult caregivers are impacted on other aspects of the social life, as they have less time to interact in social settings and this also might impact their family relationships and dynamics. Financial insecurities also abound for older caregivers who may have to work less and spend more [123]. Data on long-term financial impact of caregiving in the Deep South remains scarce; however, when the employment status of caregivers of Medicare beneficiaries aged 65 years and older were assessed, non-Hispanic Blacks and those living with care recipients were the least employed at 46.3% and 39.1%, respectively [120]. To address the gap in knowledge for this at risk group of older adults, research studies that details the adverse physical, mental, and social health outcomes of older adult caregivers in the Deep South is needed. The inclusion of older adult caregivers in both epidemiological and clinical research is also vital to eliminating the gap in knowledge for this group.

### Strategies and opportunities for improving the knowledge gap

Indeed, the literature in these areas highlighted above are particularly sparse as it relates to the prevention of disability and preservation of independence among older adults living in the Deep South. Here we discuss a few of the potential avenues for addressing the literature gaps in these areas.

#### Use of “Real World Data”

Opportunities for research appear to abound in this area, nevertheless, key strategies will be needed to truly advance the science in these areas and overcome specific challenges unique to the Deep South. A substantial proportion of clinical trials place an upper age limit on enrollment so that the “oldest older adults” are often excluded [124]. Concerns about unrepresentative samples remain relevant even in studies focused on the geriatric population, where up to 94% of studies exclude individuals with pre-existing cognitive impairment without sufficient justification, and only 43% of studies provide justification for other exclusion criteria [125]. Similar exclusions can occur in observational studies [126, 127]. Studies employ these inclusion / exclusion criteria to ensure safety of participants and to reduce sample heterogeneity so treatment effects can be detected [128]. However, such exclusions can significantly limit generalizability of trial results if significant segments of the population are ineligible [128]. Furthermore, exclusion criteria often disproportionately affect individuals from underrepresented groups [129].

The use of real-world data (RWD) has been proposed as a potential solution to these biases in observational studies. RWD refers to data routinely collected from a variety of sources and includes electronic health records (EHRs), administrative data, patient registries, patient-generated information outside of clinical settings (such as wearable sensors), and measures collected outside of the clinical setting that are relevant to health care—such as environmental exposures and socioeconomic indicators [130]. Within the healthcare system, EHR and administrative data are likely the most relevant and readily available, although many systems are incorporating other forms of RWD. Analysis of RWD typically encompasses large numbers of patients (an example of “big data”) with minimal selection criteria, which can improve generalizability of results. Pragmatic clinical trials have potential to reduce biases in the study of interventions [131]. Like observational studies of RWD, pragmatic trials typically utilize large patient samples with minimal selection criteria, but differ by using random assignment to treatment or control groups. Treatment assignment is often done at the unit (e.g., hospital ward) level rather than the individual level [131], promoting inclusion of a broad range of participants. Pragmatic trials frequently use routinely collected data, such as that from EHRs, as outcomes to enhance generalizability.

Although the use of RWD shows considerable promise to mitigate inequities in the study of at risk and under-resourced older adults, there are also limitations. Most notably, since RWD are collected for patient care rather than research purposes, the quality of the data may not be sufficient for rigorous analysis, particularly for conditions that were not the focus of clinical care. In addition, much of the information about patients’ clinical care is recorded as free text, which may not be included in RWD or may require human reviewers to manually review text for extraction into structured formats. Such approaches are costly and may limit the richness of clinical information extracted. Natural language processing (NLP) approaches can alleviate this problem, but their usage requires technical expertise that is often not readily available. Additionally, complex clinical concepts, including those related to social and functional disability, are poorly represented in NLP terminologies such as SNOMED CT [132, 133].

#### Dissemination and implementation science

Implementation science is the study of methods or approaches to promote the uptake of research findings and other evidence-based practices into routine clinical care [134], and uses implementation strategies (methods or techniques) to enhance the adoption, implementation, and sustainability of a clinical practice [135]. A number of implementation strategies and taxonomies have been published in the literature in relation to public health, educational, and preventative programs. For example, the Expert Recommendations for Implementing Change (ERIC) study generated 73 discrete implementation strategies, definitions, and categories to guide implementation research [136, 137]. This work has been critical for the field, as it provides common definitions and comprehensive descriptions of strategies that can be used to promote the uptake of research findings into routine clinical practice.

Although there is important literature on implementation of evidence-based practices for older adults in both the inpatient and outpatient settings, aging-focused research testing implementation strategies is limited. A recent review article by McNett et al. examined the implementation of evidence-based interventions in adult critical care settings. The most common practices reported were use of a ventilator-associated pneumonia bundle, nutritional support protocols, and the Awakening and Breathing Coordination, Delirium Monitoring/Management, and Early Exercise/Mobility bundle [138]. Interestingly, they also found that the most common strategies used to implement those clinical interventions were educational meetings, audit and feedback, developing tools, and use of local opinion leaders; they also found that 93% of the studies in the review reported using more than one implementation strategy [138].

In the outpatient setting, an example implementation effort is the Veterans Health Administration’s release of a national handbook (including program goals, inclusion/exclusion criteria, and anticipated benefits) and development of a community of practice to implement the geriatric patient-aligned care (GeriPACT) team [139]. GeriPACT is a patient-centered medical home model that serves older veterans with chronic disease, declining physical abilities, and/or challenges with their thinking or memory through a single point of contact [139]. Additionally, the World Health Organization highlights evidence-based intervention programs as an opportunity to improve programs and services to enhance health, literacy, and self-management in their Decade for Healthy Aging Proposal [140]. The evidence-based practices can provide insight on how to facilitate autonomy and choice for community-dwelling older adults. Some examples of evidence-based, community-focused programs include, 1) Living Healthy: A chronic disease self-management program; 2) Vivifrail; 3) Walk with Ease; 4) Capable; 5) Active Living Every Day; 6) Matter of Balance; and 7) Program for Encouraging Active Rewarding Lives (PEARLS) [141].

Future aging-related implementation research should focus on use of implementation strategies (discrete and multifaceted) to promote the uptake of evidence-based practices for older adults into routine care in the inpatient and outpatient setting. Callahan and colleagues recommend collaborative research strategies to advance the implementation of function in care of older adults. Examples include mobility programs, functional assessments, cognitive and sensory assessments, methods to better identify functional limitations, and the role of culture, environment, and the health system in integrating function into research [142]. In doing so, tailoring of strategies should be sensitive to local context and the patient population. For the Deep South, this includes considering issues such as under-resourced health systems, distrust of research, and a population with social determinants of health (e.g., low levels of literacy, poverty).

#### Community-engaged research

There is a plethora of evidence that health disparities exist among older African Americans in the South; the real challenge is in developing, implementing, and sustaining effective strategies to eliminate these. Community-engagement (CE) and community-engaged research are increasingly viewed as the keystone to translational medicine and improving the health of underserved communities [143]. Community-based participatory research (CBPR) is a transformative research opportunity that unites the growing interest of health professionals, academics, and communities in giving underserved communities a genuine voice in research, and therefore to increase the likelihood of success for these interventions [144, 145].

CBPR is a collaborative approach to research that equitably involves all partners (academia and community) in the research process and recognizes the unique strength that each brings [146]. In this type of research, academia and the community form a joint partnership to address community issues to address health disparities [147, 148], and bring about demonstrable positive health outcomes [149]. Core elements of CBPR include: 1) recognition of the community as a unit of identity with whom the research partners—i.e. as integral to all aspects of the process; 2) building on the strengths and resources within the community that the researchers explicitly recognize; 3) facilitation of equitable partnerships in all phases of the research, which includes a power-sharing process that focuses on equity; 4) recognition that socially and economically marginalized communities have not had the power to define their experience, and researchers should focus attention to the knowledge and expertise of community members; 5) balance between research and action for the mutual benefit of all partners; 6) emphasizing public health problems of local relevance and perspectives of the multiple determinants of health; 7) a cyclical and iterative process; 8) all knowledge generated is disseminated to all partners; 9) a commitment to long-term processes and sustainability.

Community input has been demonstrated to enhance both the quality and acceptability of interventions. CBPR has been recommended as a promising strategy for clinical research that aligns with the priorities of stakeholders as means to deliver appropriate care to underserved communities [150]. Most importantly, CBPR has been proven to reduce health disparities and is therefore a key and necessary step towards building health equity.

In the last few decades in the US, there has been increasing understanding of the need to reach community groups with health advances to address widespread health disparities [148]. As such, there has been a growth in the number of academics who practice CBPR, advocating for power-sharing and partnership that benefits underserved communities. Building on this and the acceptance of systemic racism as a key barrier to health equity, NIH has committed to funding such studies, a large majority of which involve partnerships such as CBPR [151], with other entities (e.g. PCORI, CDC) also providing support for CBPR focused studies. Additionally, given the cultural awareness history tied into the Deep South, it is also critical for researchers to also utilize a more culturally responsive and equitable evaluation (CREE) approach. CREE incorporates principles of diversity, equity, and inclusion into all aspects of research and evaluation—and goes beyond community-engaged research or CBPR.

#### Training the aging research workforce

Researchers have made greater efforts in recent years to increase diversity in clinical trials. Both the Food and Drug Administration [152] and National Institutes of Health [153] have made recommendations and guidelines for data collection and inclusion of diverse populations in clinical trials. In 2001, the NIH updated its policy to provide a minimum standard of inclusion for sex, gender and racial and ethnic minority groups in Phase 3 clinical trials, which is one of the final phases of research, showing safety and efficacy of the drug on the study participants. In 2017, the NIH announced an amendment requiring investigators to submit trial results for these specific groups to ClinicalTrials.gov [154]. From the report by Flores et al., “findings suggest that NIH policies on reporting of identified groups have increased over time, but a need to focus such policies beyond reporting to representative enrollment remains” [33]. In late February 2021, the NIH launched an initiative called “Ending Structural Racism – UNITE [155] aimed at ending structural racism and racial inequities in biomedical research.

To ensure that researchers understand the unique health problems of residents in the Deep South there is a need to recruit, train, and support diverse individuals that relate to this region—not only from a scientific perspective, but also from a demographic and societal view, given their own experiences in this milieu. To accomplish this goal, there is a need to understand the training structures that have been put in place from national funding agencies to specifically address the engagement of under-represented minority (URM) researchers, and how they often fail to meaningfully engage them. Although 13% of the population in the US is Black, Black researchers only account for 6% of all researchers in the STEM fields; despite the fact that Diversity, Equity and Inclusion (DEI) are a top NIH priority [156].

In the context of research, in 2006, the National Center for Advancing Translational Sciences (NCATS) established the national consortium of the Clinical and Translational Science Awards (CTSA). The main goal of this program was (and still is) accelerating translation of discoveries to improve human health, and from its inception aimed to create initiatives designed to develop and retain a diverse workforce [157]. In the context of mentoring, the National Research Mentoring Network (NRMN) adopted an evidence-based “best practices” approach [158], to mentoring, with a nationwide consortium of biomedical professionals and collaborating institutions, to support trainees at multiple levels across multiple scientific disciplines. The NRMN curriculum has been adapted and implemented successfully by CTSAs around the country.

Despite significant successes, funding and efforts, there is still today a persistent issue, often described as a “leaky pipeline” for the success of URM researchers [159]. Overall, URMs publish at lower rates and are less likely to be awarded R01 level funding; this may be reflective of the fact that despite all efforts by NIH, URM researchers still receive less robust mentorship, which is a key factor on enhancing workforce development [156]. Existing barriers include lack of meaningful mentorship when it is delivered from a distance, lack of understanding of “extra academic” stressors in the workplace, and lack of training for mentees around self-efficacy combined with cultural-sensitivity to feel more confident in their roles as researchers [160–162]. Initial inroads have been made by entities such as the Deep South Network for Translational Research to form regional relationships among multiple institutions [163]; however significant work remains to strengthen these relationships as well as to provide collaborative training that focuses on addressing issues of aging and independence.

## Conclusion

In summary, the Deep South is a region of the U.S. with a heavy burden of poverty, a long history of cultural discrimination including systemic racism, and residents with comparatively poor health including significantly elevated rates of chronic disease. A growing number of published studies now details significant health disparities in the Deep South by various characteristics including race; however, surprisingly little is known specifically for the population of this region regarding healthy aging, particularly on the context of maintenance of independence. Therefore, substantial research is needed to further characterize the disability burden among older adults in the Deep South, and to create efficacious interventions tailored to the unique aspects of individuals and communities in the region. While no single review can capture the myriad of issues at need of future research, our hope is that this work will contribute to fostering conversation and needed work in this direction to meet the substantial health needs of older adults in the Deep South region.

## Data Availability

Not applicable.
